# Prolonged corrosion protection via application of 4-ferrocenylbutyl saturated carboxylate ester derivatives with superior inhibition performance for mild steel

**DOI:** 10.1038/s41598-024-64471-0

**Published:** 2024-06-15

**Authors:** Hajar Jamali, Saleh Moradi-Alavian, Elnaz Asghari, Mehdi D. Esrafili, Elmira Payami, Reza Teimuri-Mofrad

**Affiliations:** 1 Organic Synthesis Research Laboratory, Department of Organic Chemistry, Faculty of Chemistry, University of Tabriz, Tabriz, Iran; 2 Electrochemistry Research Laboratory, Department of Physical Chemistry, Faculty of Chemistry, University of Tabriz, Tabriz, Iran; 3 Laboratory of Theoretical Chemistry, Department of Chemistry, University of Maragheh, Maragheh, Iran

**Keywords:** Ferrocene, Esterification, Electrochemical impedance spectroscopy, Corrosion inhibition, Organic chemistry, Chemical synthesis, Corrosion

## Abstract

A series of 4-ferrcenylbutyl carboxylate esters with different alkyl chain length (C_2_-C_4_) of carboxylic acids were synthesized using Fe_3_O_4_@SiO_2_@(CH_2_)_3_-Im-bisEthylFc[I] nanoparticles as catalyst and have been characterized with FT-IR, ^1^H NMR, and ^13^C NMR. Ferrocenyl-based esters were used as corrosion inhibitors of mild steel in the 1M HCl solution as corrosive media. The corrosion inhibition efficiency of the synthesized ferrocenyl-based esters has been assessed by electrochemical impedance spectroscopy (EIS), potentiodynamic polarization (PDP), atomic force microscopy (AFM), and scanning electron microscopy (SEM). The 4-ferrocenylbutyl propionate showed a more effective corrosion inhibition behavior among the studied esters with 96% efficiency after immersion in the corrosive media for 2 weeks. The corrosion inhibition mechanism is dominated by formation of passive layer of inhibitor on the surface of the mild steel by adsorption. Moreover, the adsorption characteristics of 4-butylferrcenyl carboxylate esters on mild steel were thoroughly explored using density functional theory calculations. It was found that the Fe atoms located around the C impurity in the mild steel are the most efficient and active sites to adsorb 4-butylferrcenyl carboxylate esters.

## Introduction

Steel and its alloys are common and efficient materials used in industrial machinery production^1,2^. Mild steel corrosion during an acidic wash is convenient damage that perturbs industrial production lines and contains serious outcomes such as technical and financial issues^3,4^. Corrosion inhibition materials have a considerable share of confronting against steel corrosion^5^. Organometallic materials are used widely for corrosion inhibition^6–8^. Some organometallic components have privileges for their distinguishable corrosion inhibition behavior, such as ferrocene and its derivatives introduced as corrosion inhibition materials^9–12^. The studies show that organic compound offer significant advantages in inhibiting the corrosion of mild steel^13–15^. Their adsorption behavior, film-forming properties, mixed-type inhibition, and compatibility with the adsorption isotherm make them important candidates for corrosion protection strategies. Understanding the structure–property relationships of these compounds through computational approaches further enhances the development of efficient corrosion inhibitors.

Investigations show that ferrocene derivatives such as alkylated ferrocene halides provide considerable corrosion inhibition properties. Halide derivatives of ferrocene may cause electrochemical perturbations in certain conditions, such as unfavorable halide coordination in copper included systems^16,17^. Ferrocene with other functional groups may be preferable rather than halides. On the other hand, esters are effectively used in corrosion inhibition or steel coating^18–20^. Accordingly, ferrocene-based esters may be used as favorable alternatives for corrosion inhibition of mild steel^21,22^.

The 4-chlorobutylferrocene has been used as the starting material for the synthesis of 4-ferrocenylbutyl-based esters^23,24^. Preparation of 4-ferrocenylbutyl-based esters is challenging when ferrocene is used as the initial reagent, and 4-chlorobutylferrocene is the first product for ester preparation. Also, the efficiency of the reaction is negligible. A larger halide would be favorable since it contains a two-step Friedel–Crafts acylation reaction and nucleophilic substitution reaction (S_N_2)^25^. Chlorine is not the best leaving functional group for this type of reaction in comparison with the better leaving functional groups such as bromine or iodine for a S_N_2 substitution reaction that are more expensive and requires harder reaction condition for high yield^26,27^. In this respect, the 4-ferrocenylbutyl iodide could be used to prepare 4-ferrocenylbutyl-based esters.

In our previous paper, the effect of various functional groups attached to the carbonyl group of the 4-ferrocenylbutyl carboxylate were investigated and results show that the saturated alkyl groups might be more effective in the corrosion inhibition of the mild steel^28^. However, there is no investigation about the 4-ferrocenylbutyl carboxylate with saturated carboxylic acids and there is no information about the mechanism of the mild steel corrosion inhibition of these compounds. Also, optimized synthesis of these inhibitors with significant yield is not available yet. In the present work, 4-ferrocenylbutyl-based saturated carboxylate esters with different alkyl chains have been investigated. These ferrocene-based esters have been synthesized by nucleophilic substitution reaction (S_N_2) of appropriate carboxylic acids with 4-ferrocenylbutyl iodide as the initial reagent in the presence of Fe_3_O_4_@SiO_2_@(CH_2_)_3_-Im-bisEthylFc[I] nanoparticles as catalyst and K_2_CO_3_ as base. The ^1^H NMR, ^13^C NMR, FT-IR spectroscopies, elemental analysis, and cyclic voltammetry (CV) have been used to characterize the synthesized ferrocene-based esters. The corrosion inhibition properties of the synthesized ferrocene-based esters have been investigated with electrochemical impedance spectroscopy (EIS), Tafel polarization, and scanning electron microscopy (SEM).

## Experimental

### Chemicals for synthesis

Synthesis grade reagents and chemicals were purchased from Merck, Sigma Aldrich, Fluka and Scharlau companies. The utilized solid reagents were used without further purification. The used solvents were purified with standard lab instructions. Ultra-pure double distilled water with conductance below 1 μScm^−1^ is used in the procedures. Argon atmosphere has been applied to provide inert reaction media. All of the glassware has been dried in an oven before use.

### Synthesis of Fe_3_O_4_@SiO_2_@(CH_2_)_3_-Im-bisEthylFc[I]

The Fe_3_O_4_@SiO_2_@(CH_2_)_3_-Im nanoparticles (I) and *bis*-EthylFc iodide (II) have been prepared according to our previous work^29^. The Fe_3_O_4_@SiO_2_@(CH_2_)_3_-Im nanoparticles has been charged into a flask with dried toluene and dispersed for 30 min until formation of homogeneous mixture. The *bis*-EthylFc iodide has been added to the solution proportionally and refluxed for 48 h equipped with mechanical stirrer at 95 °C. The general rout for prepared catalyst is given in Fig. S1 in supporting information. Afterward, the Fe_3_O_4_@SiO_2_@(CH_2_)_3_-Im-bisEthylFc[I] (III) catalyst have been separated using a magnet, washed with dried toluene, and characterized with FT-IR, SEM, and EDX analysis. Consequently, the prepared catalyst has been used for preparation of 4-butylferrocene carboxylate esters.

### Synthesis of 4-ferrocenylbutyl carboxylate esters

Ferrocene is used as starting material to synthesize 4-ferrocenylbutyl saturated carboxylate esters according to our previous experiences (Fig. S2)^29,30^.

The synthesized 4-ferrocenylbutyl acetate, propionate and butyrate **6a-c** have been characterized with FT-IR (Bruker Tensor 270), ^1^H NMR (Bruker, 400 MHz), ^13^C NMR (Bruker, 100 MHz), and elemental analysis (Analytik Jena Novaa 400 AAS—Elementor Vario ELIII). The results are available from our previous published work^30^.

Cyclic voltammetry (CV) has been implemented using Metrohm Autolab (PGASTAT 30) as an extra characterization for the synthesized ferrocene-based esters **6a-c**. The setup of the measurement contained a glassy carbon electrode with a 2 mm diameter (working electrode), Ag/AgCl (reference electrode), and platinum wire (counter electrode). Lithium perchlorate was used as a supporting electrolyte with a concentration of 0.1 M in acetonitrile. Also, 1 mM of ferrocene-based ester has been utilized to perform CV measurement with different scan rates^31^.

### Preparation of metallic samples for corrosion studies

The 1 × 1 cm^2^ mild steel (low carbon steel) sheets were used as subjective material for corrosion study in 1 M HCl aqueous solution as corrosive media^32^. The working electrode utilized in the experiment was composed of mild steel. Its chemical composition, expressed in weight percentage, included the following elements: 1.25% silicon (Si), 0.221% manganese (Mn), 0.021% chromium (Cr), 0.020% sulfur (S), 0.010% phosphorus (P), 0.050% carbon (C), and the remaining portion was iron (Fe). It should be noted that the pretreatment of the mild steel has been carried out using wet 500, 1500, and 2500 grade SiC papers polishing to obtain a fresh surface of the mild steel samples, and they are washed with distilled water before further processing. The synthesized 4-ferrocenylbutyl carboxylate esters (**6a-c**) with different alkyl chains (C_2_-C_4_) has been used as corrosion inhibition materials with 1, 2, 4, and 8 mM concentrations in the mentioned corrosive media.

### EIS and potentiodynamic polarization (PDP) measurements

All electrochemical measurements were carried out in a three-electrode configuration at room temperature; the mild steel sheets, Pt plate, and Ag/AgCl (3 M KCl) electrodes were working, counter, and reference electrodes, respectively. The working electrode was immersed in the corrosive solution for 120 min. Prior to the EIS studies. The EIS measurements were then performed at OCP in a frequency range of 10 kHz to 10 mHz with an AC voltage of ± 10.0 mV. An AUTOLAB PGSTAT30 equipped with a Frequency response analyzer, FRA, module was utilized for this purpose. The Tafel polarization was also performed after EIS measurements in the potential range of − 0.25V to + 0.25V vs OCP and a scan rate of 0.001 V/s^33,34^.

### DFT computations details

To simulate the adsorption behaviour of 4-butylferrcenyl carboxylate esters onto mild steel, spin-polarized DFT calculations were performed utilizing the dispersion-corrected PBE^35^ functional and a double-numerical basis set augmented with the polarization functions (DNP). The DFT semicore pseudopotentials (DSPPs)^36^ method was used to handle the relativistic effects caused by the core electrons of the Fe atom. To address the dispersion effects, Grimme’s DFT-D2^37^ technique was utilized. The basis set cut-off was 4.6 Å, and a Fermi smearing value of 0.005 Ha was used in the DFT computations.

A carbon impurity was introduced into the Fe (100) surface to simulate mild steel. To eliminate interactions between periodic pictures, a vacuum space of 15 Å was set in the direction normal to the surface. A 3 × 3 × 1 Monkhorst–Pack grid was used to sample the Brillouin zone integration. Adsorption energies (*E*_ads_) were calculated using the following equation to determine the strength of the interaction between the 4-butylferrcenyl carboxylate esters and the mild steel:

1 $$E_{{{\text{ads}}}} = E_{{{\text{comp}}}} {-}E_{{\text{X}}} {-}E_{{{\text{steel}}}}$$

where *E*_X_ and *E*_steel_ are the total energies of the isolated 4-butylferrcenyl carboxylate ester and mild steel, respectively, while *E*_comp_ is the total energy of formed complex between them. Hence, exothermic adsorption should result in a negative *E*_ads_ value. Charge-transfer effects were analysed by the Hirshfeld method. All the calculations were performed using the DMol^3,38^ electronic structure code.

### Morphology changes during corrosion of mild steel samples

The scanning electron microscopy (MIRA3 FEG-SEM) was used to inspect the surface of the mild steel and the effect of the studied corrosive media in the presence of the synthesized 4-ferrocenylbutyl carboxylate esters with different alkyl chains (C_2_-C_4_). The mild steel surface has been inspected with SEM before and after 24 h static stable corrosion media immersion^33^.

## Results and discussion

### Synthesis of Fe_3_O_4_@SiO_2_@(CH_2_)_3_-Im-bisEthylFc[I]

The prepared nanoparticles have been characterized using FT-IR, SEM, and EDX analysis that the results are given in Fig. S3. The results show that the procedure for preparation of the Fe_3_O_4_@SiO_2_@(CH_2_)_3_-Im-bisEthylFc[I] (III) was successful. The prepared nanomaterial has been used as catalyst in the synthesis of the 4-ferrocenylbutylcarboxylate as described in the next section.

### Synthesis of 4-ferrocenylbutyl-based carboxylate esters

The esterification reaction of 4-iodobutylferrocene in the presence of catalyst (III) has been implemented to prepare 4-ferrocenylbutyl carboxylate esters. The catalyst (III) was prepared similar to the our previous work^29^. The Friedel–Crafts acylation reaction of the ferrocene was implemented to obtain 4-chlorobutyroylferrocene (2). Subsequently, 4-chlorobutylferrocene (3) was synthesized with insitu reduction of compound 2 with the solution of NaBH_4_ in diglyme^31^. Besides, the halogen substitution was conducted according to the literature to achieve 4-iodobutylferrocene (4). The mixture of compound 4, appropriate carboxylic acids (5a-c), and the nanoparticles III as catalyst were used to synthesize the ferrocene-based esters (6a-c) in the presence of potassium carbonate (K_2_CO_3_) as base and CH_3_CN as the solvent^30^. The modified reaction with the Fe_3_O_4_@SiO_2_@(CH_2_)_3_-Im-bisEthylFc[I] as catalyst improved the reaction rate and reduced required time for finalizing the esterification reaction of 4-iodobutylferrocene compared to our previous work^30^. The obtained results of synthesis of 4-ferrocenylbutyl carboxylate esters with different alkyl chains (C_2_-C_4_) were showed in Table S1.

The CV voltammograms of the esters are compared in Fig. S4 for different scan rates. The corresponding obtained parameters such as anodic peak current (I_p,a_), cathodic peak current (I_p,c_), baseline corrected anodic to cathodic peak current ratio (I_p,a_ /I_p,c_), and absolute difference of anodic peak potential (E_p,a_) and cathodic peak potential (E_p,c_), |E_p,a_-E_p,c_| are collected in Table S2. The results show only one electroactive component in the studied system. The observed electroactivity is due to the ferrocene group^39^.

### Potentiodynamic polarization

**Figure 1:**
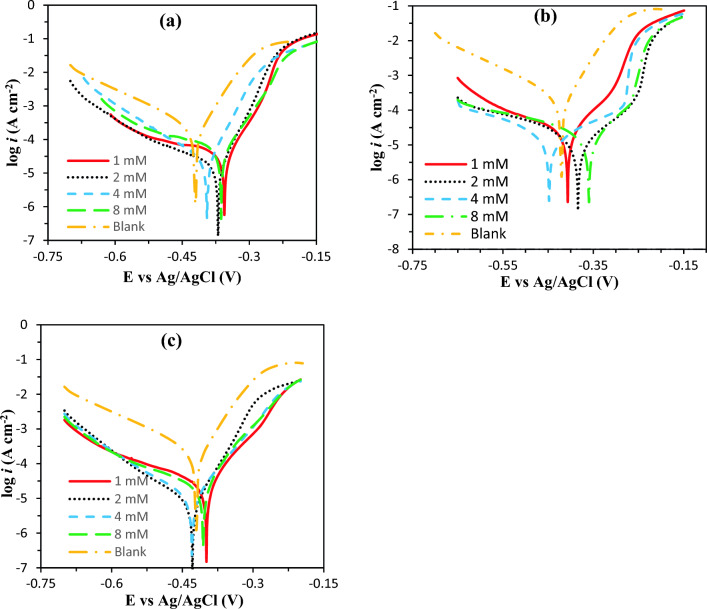
The polarization curve of the mild steel in 1 M HCl in the presence of different concentration: (**a**) **6a**, (**b**) **6b**, and (**c**) **6c**.

The polarization curve of the mild steel without and with synthesized 4-ferrocenylbutyl carboxylates inhibitors has been shown in Fig. 1.

Inhibitor’s oxidation and reduction reactions in the acidic media are controlled by adding inhibitors due to decreasing the dissolution rate of mild steel and restricting the hydrogen evolution reactions. Polarization parameters such as corrosion potentials (*E*_*corr*_), corrosion current densities (*I*_*corr*_), cathodic and anodic tafel slopes (*β*_*a*_ and *β*_*c*)_, and the polarization resistance (*R*_*p*_) were obtained by extrapolating the linear parts of the polarization diagrams and the relevant data were given in Table 1.

**Table 1:** Polarization parameters of mild steel immersed in 1 M HCl in absence and presence of different inhibitors concentrations.

Immersion time (h)	C_inh_ (mM)	*E*_*corr*_ (mV vs. Ag/AgCl)	*I*_*corr*_ (µA/cm^2^)	*Ba* (mV/dec)	*Bc* (mV/dec)	*R*_*p*_ (Ω cm^2^)	*η* (%)
Blank							
2	–	− 421.10	152.45	46.67	122.26	96.22	–
**6a**							
2	1	− 394.06	18.45	39.44	101.41	668.28	85.60
2	2	− 368.82	15.12	39.92	174.11	932.71	89.68
2	4	− 355.56	27.26	48.60	163.11	596.48	83.87
2	8	− 363.16	41.09	52.85	181.33	432.54	77.75
**6c**							
2	1	− 398.76	20.51	64.87	142.49	944.04	89.81
2	2	− 428.62	6.71	46.05	118.14	2144.70	95.51
2	4	− 429.55	10.28	64.17	126.43	1498.10	93.58
2	8	− 405.99	17.23	53.06	155.98	998.32	90.36
**6b**							
2	1	− 406.70	20.61	86.39	164.99	1195.00	91.95
2	2	− 383.79	8.11	98.21	109.80	2771.70	96.53
2	4	− 447.76	9.53	123.18	115.88	2720.10	96.46
2	8	− 359.86	12.16	82.48	152.55	1911.60	94.97
170	2	− 387.19	9.31	108.28	133.09	2786.10	96.54
340	2	− 415.46	15.69	74.09	131.72	1312.90	92.67

Inhibition efficiency of the inhibitors was calculated using the Eq. (2):

2 $${\varvec{\eta}}\left(\boldsymbol{\%}\right)=\frac{{{\varvec{R}}}_{{\varvec{p}}\left({\varvec{i}}{\varvec{n}}{\varvec{h}}\right)}-{{\varvec{R}}}_{{\varvec{p}}}}{{{\varvec{R}}}_{{\varvec{p}}\left({\varvec{i}}{\varvec{n}}{\varvec{h}}\right)}}$$

where *R*_*p(inh)*_ and *R*_*p*_ represent the polarization resistance with and without corrosion inhibitor, respectively. Stern-Geary equation was applied to calculate the polarization resistances, according to Eq. (3):

3 $${{\varvec{R}}}_{{\varvec{p}}}=\frac{{{\varvec{\beta}}}_{{\varvec{c}}}{{\varvec{\beta}}}_{{\varvec{a}}}}{2.303\boldsymbol{ }{{\varvec{I}}}_{{\varvec{c}}{\varvec{o}}{\varvec{r}}{\varvec{r}}}({{\varvec{\beta}}}_{{\varvec{c}}}+{{\varvec{\beta}}}_{{\varvec{a}}})}$$

Based on the data listed in Table 1, *I*_*corr*_ decreased in the presence of inhibitors and the most significant effect was observed at 2mM of the inhibitors. It is generally attributed to the adsorption of inhibitors on the surface of the metal. The *E*_*corr*_ values did not show significant changes with inhibitor concentration in a potential range of less than 85 mV indicating a mixed corrosion control of the used inhibitors^40–43^. The *β*_*a*_ and *β*_*c*_ also did not change significantly that confirms the mixed controlled corrosion of the inhibitors. Forming a protective layer on the surface of metal, inhibitors decrease the *I*_*corr*_ and increase *R*_*p*_ respectively over almost 10 and 20 times in their optimized concentrations. Among inhibitors, 6b inhibitor showed the best performance with 96.53% inhibition efficiency after 2 h immersion in 1 M HCl.

### Electrochemical impedance spectrums

**Figure 2:**
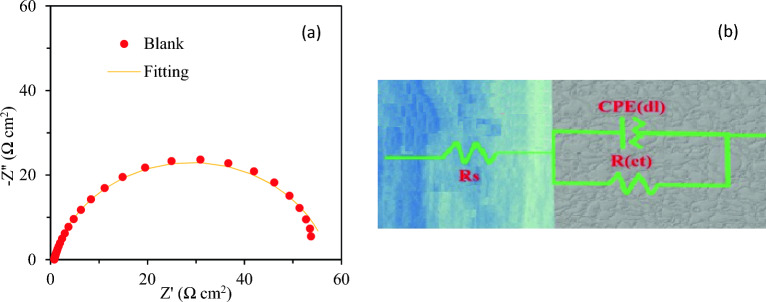
(**a**) The Nyquist plot of mild steel in 1 M aqueous HCl at 25 °C with corresponding fitting curve and (**b**) the equivalent circuit for analysis and fitting.

The Nyquist plot of mild steel in 1 M HCl at 25 °C is given in Fig. 2a. The presence of only one semicircle in Nyquist plot confirms a simple Randles cell model with a solution resistance, *R*_*s*_, in series with a parallel-connected charge transfer resistance, *R*_*ct*_, and constant phase element, CPE (Fig. 2b). The Nyquist plots of the mild steel in inhibited HCl solutions have also been presented at Fig. 5. The protective inhibitor film on the surface of mild steel resulted in a deformed semicircle; Refs.^44,45^ based on the fitting results such a semicircle did not fit properly to the equivalent circuit model shown in Fig. 2a.

**Figure 3:**
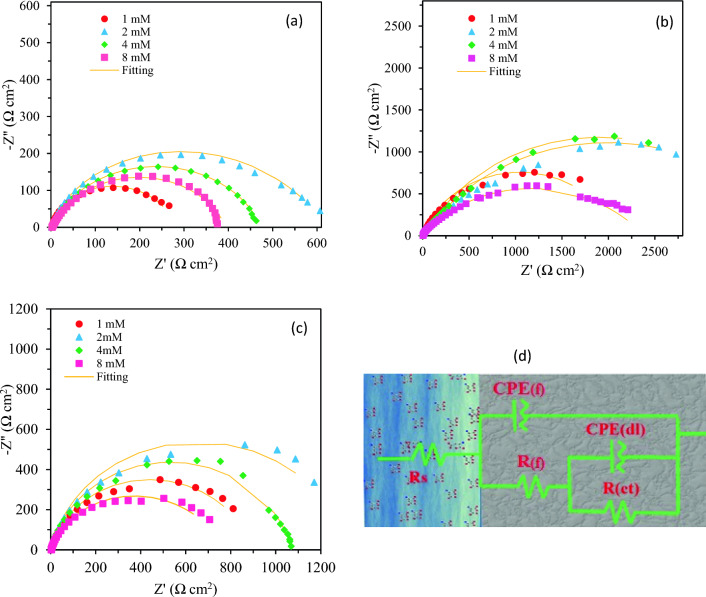
The Nyquist plot of the mild steel in 1 M aqueous HCl media in the presence different concentration of: (**a**) **6a**, (**b**) **6b**, (**c**) **6c**, and (**d**) the equivalent circuit used for fitting.

The behaviour of inhibited samples was best fitted to a two time-constant model shown in Fig. 3d. In this model the film resistance, *R*_*f*_, and the corresponding *CPE* (*CPE*_*f*_) are used to simulate the additional inhibitor film related high frequency time constant. *CPE* is a general element that is used to describe the behaviour of a non-ideal capacitance and the admittance Y serves as the basis for defining the CPE, which is determined using Z_CPE_ = (Y_0_ ω j)^−n^, where Y_0_ and n represent the relevant admittance and power of the CPE, respectively, while ω denotes the angular frequency and j the imaginary number.

The capacitance values were calculated according to Eq. (4)^11^:

4 $${C}_{dl}={(Y}_{0}{R}_{ct}^{1-n}{)}^{1/n}$$

The resulted parameters obtained from fitting the EIS experimental data are given in Table 2 with the corresponding fitting errors.

**Table 2:** The corrosion inhibition efficiency of the 4-ferrocenylbutyl carboxylate esters with corresponding fitting parameters for EIS data.

Immersion time (h)	C_inh_ (mM)	CPE_f_		R_f_ (Ω cm^2^)	CPE_dl_		R_ct_ (Ω cm^2^)		Η (%)	Fitting error
		Y_0_ × 10^−6^ (Ω^−1^ cm^−2^S^n^)	n		Y_0_ × 10^−6^ (Ω^−1^ cm^−2^S^n^)	n		C_dl_ (µF cm^−2^)		
Blank										
2	–	–	–	–	143.61	0.87	57	70.04	–	0.0014
**6a**										
2	1	34.34	0.97	5	100.08	0.74	277	28.39	79.42	0.0005
2	2	29.52	0.95	24	146.58	0.59	602	27.13	90.53	0.0023
2	4	31.20	0.93	20	149.56	0.66	466	37.92	87.77	0.0015
2	8	34.77	0.85	33	80.76	0.79	361	31.56	84.21	0.0004
**6c**										
2	1	41.21	0.90	94	14.83	0.73	977	3.10	94.17	0.0023
2	2	33.48	0.91	104	22.42	0.63	1225	2.71	95.35	0.0032
2	4	36.69	0.91	50	38.03	0.62	855	4.66	93.33	0.0019
2	8	28.17	0.92	46	79.02	0.59	743	11.02	92.33	0.0020
**6b**										
2	1	29.42	0.77	22	18.20	0.95	2076	15.32	97.25	0.0019
2	2	2.76	0.92	127	41.43	0.61	3884	12.88	98.53	0.0067
2	4	5.99	0.91	180	41.14	0.66	3656	15.50	98.44	0.0041
2	8	2.52	0.90	136	70.93	0.53	2254	13.96	97.47	0.0026
170	2	5.45	0.80	573	84.03	0.53	1753	15.37	96.75	0.0030
340	2	92.47	0.74	267	58.51	0.75	1394	25.37	95.91	0.0014

Inhibition efficiency of the inhibitors was calculated with the Eq. (5):

5 $${\varvec{\eta}}\left(\boldsymbol{\%}\right)=\frac{{{\varvec{R}}}_{{\varvec{c}}{\varvec{t}}\left({\varvec{i}}{\varvec{n}}{\varvec{h}}\right)}-{{\varvec{R}}}_{{\varvec{c}}{\varvec{t}}}}{{{\varvec{R}}}_{{\varvec{c}}{\varvec{t}}\left({\varvec{i}}{\varvec{n}}{\varvec{h}}\right)}}$$

In this equation, the *R*_*ct(inh)*_ and *R*_*ct*_ are the charge transfer resistances in the presence of inhibitor and without inhibitor, respectively. The protective layer results in a decrease in the rate of charge transfer (electron exchange) between metallic surface and the electrolyte and causes an increase in inhibition efficiency^33,46,47^. Thus, a significant increase in *R*_*ct*_ and a remarkable decrease in *C*_*dl*_ values in the inhibited solutions compared with the values for samples corroded in blank are observed in Table 2. The reduced *C*_*dl*_ values for inhibited samples is attributed to the increase in the thickness of the double layer capacitor and the replacement of water molecules with the inhibitor molecules, confirming the adsorption of inhibitor molecules on metal^11,48^. The values of power of *CPE*_*f*_ (*n*_*f*_) are very close to 1 and show the near-ideal capacitance behaviour of the adsorbed inhibitor film; it is also due to the homogeneous distribution of activation energies on the surface of the inhibitive film resulting from an almost homogeneous adsorption of the inhibitor molecules on metal surface. The *R*_*f*_ values showed a remarkable increase with concentration up to a maximum and the slight decrease was seen generally due to the changes in inhibitive film thickness and structure. The n values for double layer related *CPE* decreased for inhibited samples representing an enhancement in the diffusion contribution in the resistance against charge transfer process through the inhibitor adsorbed layer. Generally, as n becomes closer to 0.5 the process is likely to be controlled by diffusion. Maximum η% values were observed for samples inhibited using 2.0 mM of each inhibitor; although their values did not change significantly for 6b and 6c with their concentration as is obvious from data in Table 2. As it is, the inhibitor 6b shows the maximum corrosion inhibition efficiency among the studied 4-ferrocenylbutyl carboxylates inhibitors. Among synthesized 4-ferrocenylbutyl carboxylates with different alkyl chains the best was observed for the samples with propionate alkyl chain (6b). The length of alkyl chain and steric hindrance are two main factors that can affect the inhibition performances. Longer alkyl chains give more effective inhibition properties^11,49^. Besides, increasing steric hindrance lowers the protective properties of inhibitors. Therefore, in designing these structures and optimized chain length and steric hindrance are expected to have the highest inhibition performance. The compound 6c with the longest chain length has the most remarkable hindrance and inhibitor 6a has the lowest alkyl chain length with the lowest inhibition efficiency among all of them. The compound 6b with an optimized structure can be adsorbed more efficiently showing the highest inhibition efficiencies.

### Immersion time and efficiency of corrosion inhibition performance

**Figure 4:**
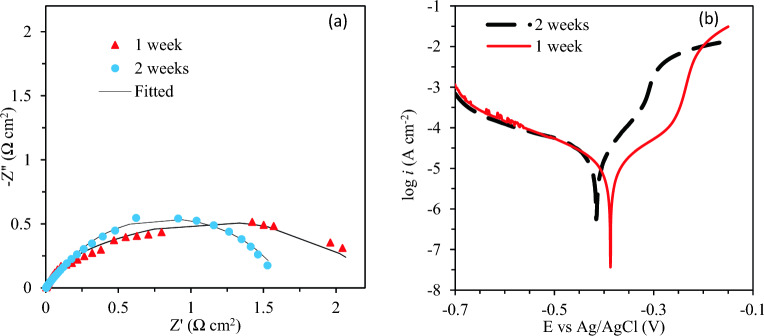
The effect of different immersion time of mild steel in 1 M HCl media in the presence 2 mM **6b** inhibitor: (**a**) the Nyquist plot and (**b**) Tafel polarization.

The inhibitor **6b** was the most efficient corrosion inhibitor among the studied ones based on the results. The effect of immersion time on the corrosion inhibition efficiency of the synthesized product inhibitor **6b** has been studied after 170 and 340 h immersion of mild steel in the 1 M HCl in the presence of the inhibitors. Accordingly, the Tafel polarization and EIS measurements have been carried out to evaluate the immersion time effect. The corresponding EIS and Tafel polarization results are given in Fig. 4 and the data presented in Table 1 and 42 It is clear that the inhibition efficiency of **6b** indicated negligible reduction in inhibition efficiencies at elevated immersion times. After 2 weeks, inhibition efficiency of **6b** inhibitor was 95.91%. As is discussed earlier, the created passive layer film prevents corrosion. *C*_*dl*_ after one week negligible increased that is indicate inhibitors molecules slightly replacement by water molecules. The uptake of water and electrolyte ions inside the adsorbed layer can also result in an enhanced conductivity and decreased *R*_*ct*_ values at elevated immersion times. Such a significant decrease in *R*_*f*_ and *C*_*f*_ at 340 h can be attributed to the risen conductivity of the inhibitive film and decreased dielectric constant.

### Morphology studies for the metal surface

**Figure 5:**
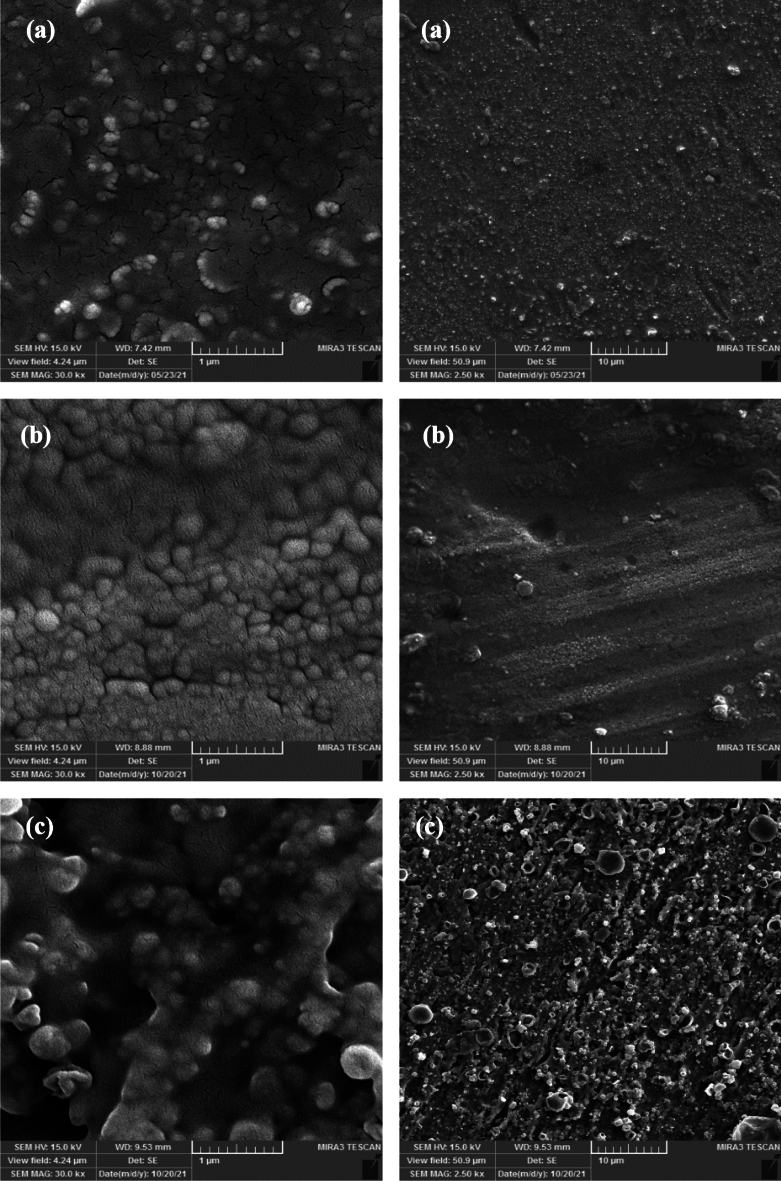
The surface of the mild steel after 24 h immersion in the HCl media with a concentration 1 M at 25 °C in the presence 2 mM of: (**a**) **6a**, (**b**) **6b** and (**c**) **6c**.

The SEM images of the mild steel after 24 h immersion in acidic media in the absence of the corrosion inhibitors have been depicted in Fig. S5. Pitting on the surface of the steel is the leading corrosion type. The scale of the created cavity in the surface was about 2 μm at this immersion time. The corrosion inhibition effect of the synthesized 4-ferrocenylbutyl carboxylate esters on the mild steel surface has been inspected with the SEM images in 1 and 10 μm scales and given in Fig. 5. As evident, the 4-ferrocenylbutyl propionate (6b) was more efficient in the corrosion inhibition rather than the other studied 4-ferrocenylbutyl carboxylate esters. Also, the figure shows the formation of a protection layer on the surface of the mild steel in the presence of the inhibitors. The observed images are in good agreement with the EIS and Tafel polarization results. According to SEM images, a multiple layer was formed on the metal substrate, so it is not possible to calculate the thermodynamic parameters.

**Figure 6:**
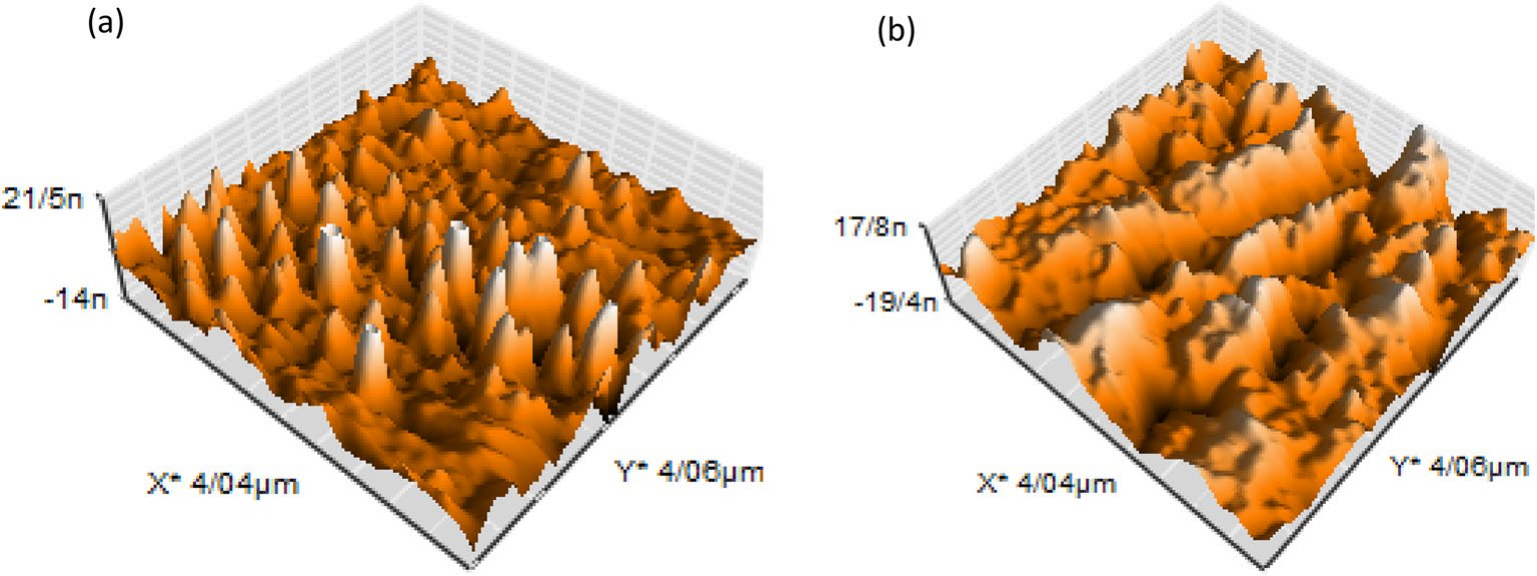
AFM images of the mild steel after 24 h immersion in the HCl 1 M (**a**) absence of inhibitor and (**b**) presence of 2 mM 6b inhibitor.

The AFM analysis of mild steel in presence and absence of **6b** inhibitor are depicted in Fig. 6. The smooth surface of mild steel in presence of inhibitor can be observed. The roughness data including Rp, Ry, Rq, and Ra which are maximum profile peak height, maximum profile valley depth, root mean square (RMS) roughness, and average roughness are represented in Table 3.

**Table 3:** Surface roughness parameters of the mild steel after 24 h immersion in the HCl 1 M in absence and presence of 2 mM **6b** inhibitor.

samples	Ra (nm)	Rq (nm)	Rv (nm)	Rp (nm)
bare	5.10	7.22	39.05	25.00
6b inhibitor	3.58	4.56	24.44	11.62

According to AFM data, in presence of **6b** inhibitor a smooth surface was obtained and surface of mild steel covered by inhibitor that is indicate formation of protective layer on the surface of metal^50,51^. The corrosion of mild steel in absence of inhibitor caused to increase surface roughness parameters values^1^.

### DFT results

**Figure 7:**
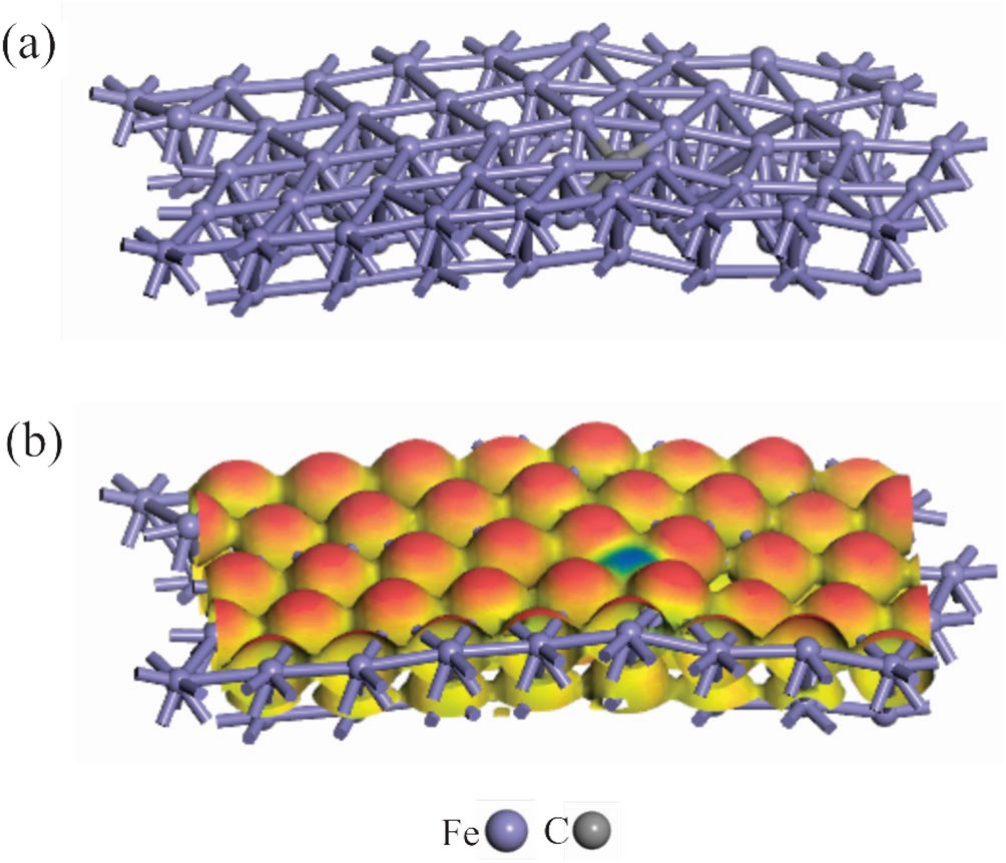
(**a**) DFT optimized geometry and (**b**) MEP isosurface (isovalue = 0.20 a.u.) of the mild steel model used in the present study. The most negative and most positive potentials in the MEP map are shown by blue and red regions, respectively.

DFT calculations can offer helpful information about the surface reactivity of mild steel towards 4-butylferrcenyl carboxylate esters. To achieve this goal, dispersion-corrected DFT calculations were used to study how the 4-butylferrcenyl carboxylate esters interact with the active sites on mild steel. First, consider the optimized structure of mild steel discussed here. The top and side views of optimized mild steel, as well as its molecular electrostatic potential (MEP), are shown in Fig. 7. It is seen that the introduction of a carbon impurity causes some structural deformation in the Fe surface (the atomic ratio of iron to carbon is 63:1), but the surface remains planar after full geometry relaxation. The latter is due to the difference in atomic radii between Fe and C atoms (1.40 versus 0.70 Å). Furthermore, the difference in electronegativity between C and Fe atoms produces a nonuniform distribution of atomic charges in the mild steel. Indeed, the MEP analysis indicates that the C atom in mild steel has a negative electrostatic potential, whereas those around it have a positive value (Fig. 7b). These findings clearly show that the addition of a C atom to the Fe surface considerably changes its electronic structure. The C atom in mild steel has a negative charge of − 0.14 |e|, whereas those around it have a positive charge of 0.08 |e|, according to the Hirshfeld analysis. This result, which confirms the MEP analysis discussed above, shows that the Fe atoms around the C atom might be viewed as potential sites for Lewis base interaction.

**Figure 8:**
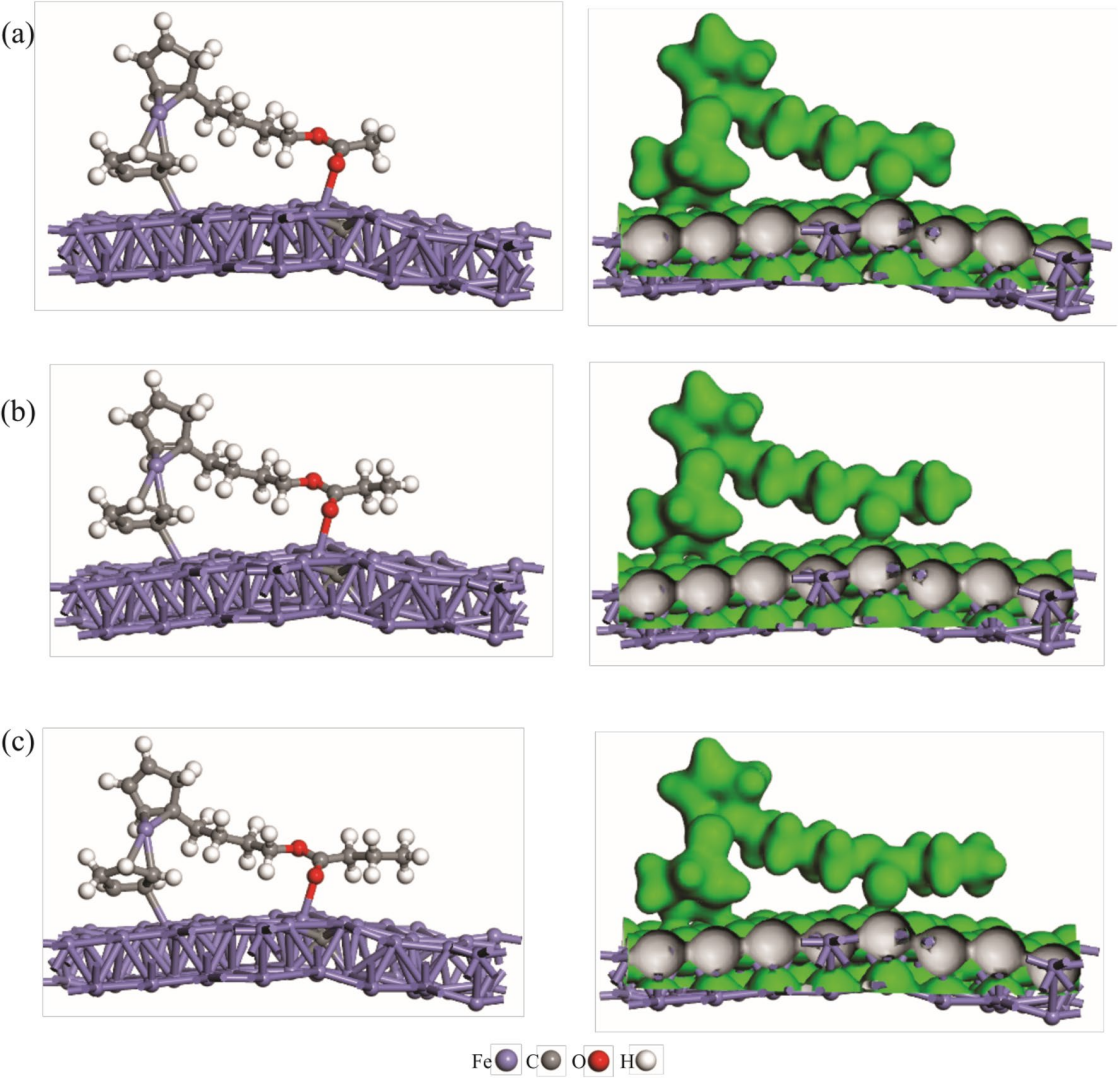
DFT optimized structures (left) and related electron density map (right, isovalue = 0.05 a.u.) of (**a**) 4-butylferrocene acetate, (**b**) 4-butylferrocene propionate and (**c**) 4-butylferrocene butyrate adsorbed onto the mild steel.

To compare the adsorption strength of 4-butylferrcenyl derivatives, these systems were allowed to interact with different regions of the mild steel individually. Various initial adsorption configurations were investigated for each system, including those in which the O atoms of adsorbates interact with the surface Fe atoms. After full geometry relaxation, it was observed that 4-butylferrcenyl carboxylate esters prefer to adsorb on the mild surface from their C=O group (Fig. 8).

Furthermore, the Fe atoms near the C impurity have a greater propensity for interacting with these systems. It should be highlighted that the cyclopentadienyl ring of esters has also a side interaction with the steel surface, as demonstrated by a rather small binding distance (≈ 2.3 Å). Table 4 reveals that the adsorption energies of 4-butylferrcenyl carboxylate esters are − 46.83, − 51.28, and − 50.08 kcal/mol, indicating that the adsorption strength of these systems does not increase with alkyl group length. This may be explained by the fact that, while the alkyl group serves as the electron-donating moiety in 4-butylferrcenyl carboxylate esters, their steric effects should also play a destabilizing role in the formation of these complexes. Thus, among the esters examined here, 4-butylferrocene propionate has the most negative adsorption energy.

**Table 4:** Calculated Fe–O binding distances (*R*_Fe-O_, Å), adsorption energies (*E*_ads_, kcal/mol) and net charge-transfer (Q_CT_, electrons) values of 4-butylferrcenyl carboxylate esters adsorbed onto the mild steel.

Adsorbate	*R*_Fe-O_	*E*_ads_	Q_CT_
4-butylferrocene acetate	2.20	− 46.83	0.14
4-butylferrocene propionate	2.19	− 51.28	0.19
4-butylferrocene butyrate	2.19	− 50.08	0.16

It is worth noting that the adsorption energies are all negative, showing that the adsorption process is exothermic and may occur spontaneously at room temperature. Some electronic charges are also expected to transfer from the 4-butylferrcenyl carboxylate esters into the steel during adsorption. As shown in Table 4, the Hirshfeld analysis demonstrates that about 0.15 electrons are shifted from the 4-butylferrcenyl carboxylate esters into the steel, with the majority of the electrons centered on the Fe atoms surrounding the C. The electron density map depicted in Fig. 8 further supports the chemisorption of the 4-butylferrcenyl carboxylate esters, which shows considerable electron density buildup near the newly formed Fe–O bond.

**Figure 9:**
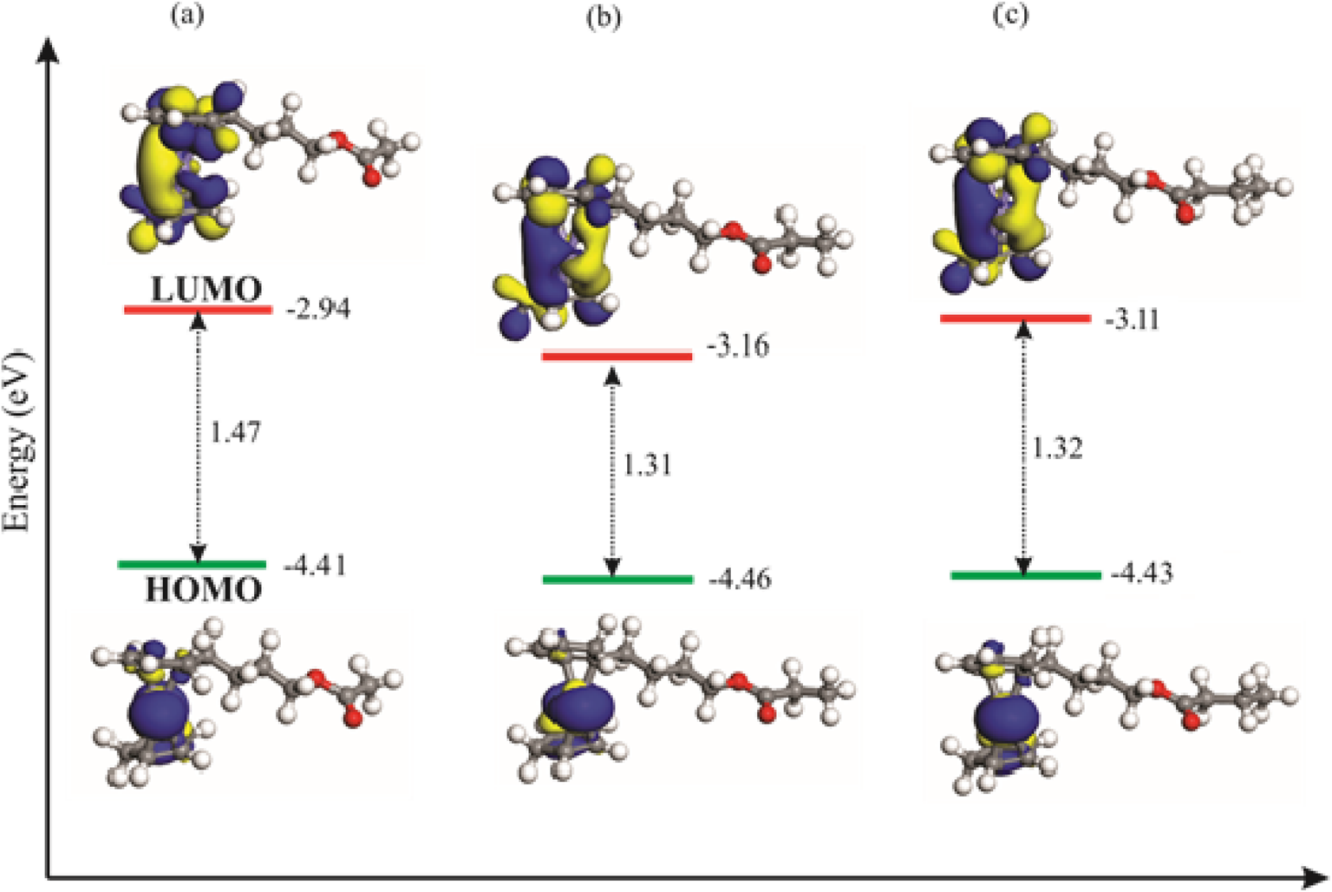
The HOMO, LUMO and energy gap values of the optimized (**a**) 4-butylferrocene acetate, (**b**) 4-butylferrocene propionate and (**c**) 4-butylferrocene butyrate molecules.

The frontier molecular orbital (FMO) analysis was performed on the 4-butylferrcenyl carboxylate esters to provide insight on their corrosion inhibition. The highest occupied molecular orbital (HOMO), lowest unoccupied molecular orbital (LUMO) and associated energy gaps of these systems are depicted in Fig. 9. The HOMO of these molecules is found to be mostly centered on the Fe atom of ferrocene moiety and has mostly d_z_^2^ character, whereas the LUMO is distributed mainly on the cyclopentadienyl rings. As the length of alkyl group increases, both the HOMO and LUMO are stabilized and their energy becomes more negative. Note that the amount of stabilization in the LUMO is larger than that of HOMO, hence the HOMO–LUMO energy gap varies as –R=–CH_3_ > –C_3_H_7_ > –C_2_H_5_. Because a lower energy gap between the HOMO and LUMO corresponds to less kinetics stability^52^, it is concluded that, in addition to its larger adsorption energy and higher charge-transfer, superior corrosion inhibition of 4-butylferrocene propionate could be related to a lower HOMO–LUMO energy gap.

## Conclusion

In the presented study, three 4-ferrocenylbutyl acetate, propionate, and butyrate esters have been synthesized and used as corrosion inhibitor of the mild steel in HCl media. In this respect, electrochemical analysis, including EIS and Tafel polarization behavior of the mild steel in the presence of the synthesized products, has been carried out. The results show that the 4-ferrocenylbutyl propionate has the best corrosion inhibition behavior among the synthesized esters with 96% corrosion inhibition efficiency. Also, it has been shown that after two-week immersion of mild steel in 1 M HCl, the efficiency of the corrosion inhibition of the 4-ferrocenylbutyl propionate is acceptable. The SEM results show that the inhibition mechanism is based on creating a passive layer on the surface of the steel. Based on the DFT calculations, the 4-butylferrcenyl carboxylate esters are chemisorbed on the Fe atom of steel located around the C impurity. The superior corrosion inhibition of 4-butylferrocene propionate can be related to its larger adsorption energy and charge-transfer as well as lower HOMO–LUMO energy gap.

### Supplementary Information

Supplementary Information.

## Data Availability

The datasets used and/or analysed during the current study available from the corresponding author on reasonable request.

## References

[1] Fernandes CM, Alvarez LX, dos Santos NE, Maldonado Barrios AC, Ponzio EA (2019). Green synthesis of 1-benzyl-4-phenyl-1H-1,2,3-triazole, its application as corrosion inhibitor for mild steel in acidic medium and new approach of classical electrochemical analyses. Corros. Sci..

[2] Roy P, Karfa P, Adhikari U, Sukul D (2014). Corrosion inhibition of mild steel in acidic medium by polyacrylamide grafted Guar gum with various grafting percentage: Effect of intramolecular synergism. Corros. Sci..

[3] Saha SK, Dutta A, Ghosh P, Sukul D, Banerjee P (2015). Adsorption and corrosion inhibition effect of Schiff base molecules on the mild steel surface in 1 M HCl medium: A combined experimental and theoretical approach. Phys. Chem. Chem. Phys..

[4] Meng Y (2017). Inhibition of mild steel corrosion in hydrochloric acid using two novel pyridine Schiff base derivatives: A comparative study of experimental and theoretical results. RSC Adv..

[5] Srivastava M (2018). Low cost aqueous extract of Pisum sativum peels for inhibition of mild steel corrosion. J. Mol. Liquids.

[6] Li W, Ren B, Chen Y, Wang X, Cao R (2018). Excellent efficacy of MOF films for bronze artwork conservation: The key role of HKUST-1 film nanocontainers in selectively positioning and protecting inhibitors. ACS Appl. Mater. Interfaces.

[7] Akbarzadeh S, Ramezanzadeh B, Bahlakeh G, Ramezanzadeh M (2019). Molecular/electronic/atomic-level simulation and experimental exploration of the corrosion inhibiting molecules attraction at the steel/chloride-containing solution interface. J. Mol. Liquids.

[8] Tian H (2018). Controlled delivery of multi-substituted triazole by metal-organic framework for efficient inhibition of mild steel corrosion in neutral chloride solution. Corros. Sci..

[9] Sayed AR, El-Lateef HMA (2020). Thiocarbohydrazones based on adamantane and ferrocene as efficient corrosion inhibitors for hydrochloric acid pickling of C-steel. Coatings.

[10] G, S., K, S., R, S. & G, R. R. Correction: The corrosion inhibition of stainless steel by ferrocene–polyoxometalate hybrid molecular materials—Experimental and first principles studies. *Phys. Chem. Chem. Phys.* **23**, 14529–14531 (2021). 10.1039/d1cp90126e 34165130

[11] Fatima S (2019). Study of new amphiphiles based on ferrocene containing thioureas as efficient corrosion inhibitors: Gravimetric, electrochemical, SEM and DFT studies. J. Ind. Eng. Chem..

[12] Morad MS, Sarhan AAO (2008). Application of some ferrocene derivatives in the field of corrosion inhibition. Corros. Sci..

[13] Sehmi A (2020). Corrosion inhibition of mild steel by newly synthesized pyrazole carboxamide derivatives in HCl acid medium: Experimental and theoretical studies. J. Electrochem. Soc..

[14] Benali, O. Corrosion inhibition of mild steel in acidic media using newly synthesized heterocyclic organic molecules: Correlation between inhibition efficiency and chemical structure. *AIP Conference Proceedings* **1653**, (2015).

[15] Ashassi-Sorkhabi H, Kazempour A (2020). Thermodynamic and kinetic insights into the role of amino acids in improving the adhesion of iota-carrageenan as a natural corrosion inhibitor to the aluminum surface. J. Adhes. Sci. Technol..

[16] Berger G, Frangville P, Meyer F (2020). Halogen bonding for molecular recognition: New developments in materials and biological sciences. Chem. Commun..

[17] Izquierdo J, Santana JJ, González S, Souto RM (2010). Uses of scanning electrochemical microscopy for the characterization of thin inhibitor films on reactive metals: The protection of copper surfaces by benzotriazole. Electrochimica Acta.

[18] Moghaddam PN, Amini R, Kardar P, Ramezanzadeh B (2021). Epoxy-ester coating reinforced with cerium (III)-tannic acid-based hybrid pigment for effective mild-steel substrate corrosion protection. Progress Org. Coat..

[19] Abdelaziz S (2021). Green corrosion inhibition of mild steel in HCl medium using leaves extract of Arbutus unedo L. plant: An experimental and computational approach. Colloids Surf. A Physicochem. Eng. Aspects.

[20] Aslam R, Mobin M, Obot IB, Alamri AH (2020). Ionic liquids derived from α-amino acid ester salts as potent green corrosion inhibitors for mild steel in 1M HCl. J. Mol. Liquids.

[21] Kilic A, Incebay H, Bayat T (2023). Experimental spectroscopic investigation and electrochemical sensor studies of facile and controllable synthesis of ferrocene-based chiral Schiff base compounds. J. Taiwan Inst. Chem. Eng..

[22] Al Kiey SA, El-Sayed AA, Khalil AM (2024). Controlling corrosion protection of mild steel in acidic environment via environmentally benign organic inhibitor. Colloids Surf. A Physicochem. Eng. Aspects.

[23] Teimuri-Mofrad R, Abbasi H, Hadi R (2019). Graphene oxide-grafted ferrocene moiety via ring opening polymerization (ROP) as a supercapacitor electrode material. Polymer.

[24] Safa KD (2014). [Tris(alkoxydimethylsilyl)methyl]alkylferrocenes as new ferrocenyl multifunctional silyl ethers. Aust. J. Chem..

[25] Otera J, Nishikido J (2009). Esterification: Methods, Reactions, and Applications.

[26] Sonego JM, de Diego SI, Szajnman SH, Gallo-Rodriguez C, Rodriguez JB (2023). Organoselenium compounds: Chemistry and applications in organic synthesis. Chem. Eur. J..

[27] Si Y, Tang P (2023). Development and application of trifluoromethoxylating reagents. Chin. J. Chem..

[28] Mirzaei-Saatlo M (2023). 4-Ferrocenylbutyl-based corrosion inhibitors for mild steel in acidic solution. Mater. Chem. Phys..

[29] Teimuri-Mofrad R, Esmati S, Tahmasebi S, Gholamhosseini-Nazari M (2018). Bisferrocene-containing ionic liquid supported on silica coated Fe3O4: A novel nanomagnetic catalyst for the synthesis of dihydropyrano[2,3-c]coumarin derivatives. J. Organomet. Chem..

[30] Jamali H, Teimuri-Mofrad R (2022). Synthesis of ferrocene-based esters by alkylation of carboxylate ions and investigation of their electrochemical and optical behaviors. Appl. Organomet. Chem..

[31] Teimuri-Mofrad R, Mirzaei F, Abbasi H, Safa KD (2017). Synthesis of new binuclear ferrocenyl compounds by hydrosilylation reactions. Comptes Rendus Chimie.

[32] Zhang GA, Hou XM, Hou BS, Liu HF (2019). Benzimidazole derivatives as novel inhibitors for the corrosion of mild steel in acidic solution: Experimental and theoretical studies. J. Mol. Liquids.

[33] Sadeghi Erami R (2019). Carboxamide derivatives as new corrosion inhibitors for mild steel protection in hydrochloric acid solution. Corros. Sci..

[34] Ashassi-Sorkhabi H, Es’haghi M (2009). Corrosion inhibition of mild steel in acidic media by [BMIm]Br Ionic liquid. Mater. Chem. Phys..

[35] Perdew JP, Burke K, Ernzerhof M (1996). Generalized gradient approximation made simple. Phys. Rev. Lett..

[36] Liu P, Rodriguez JA (2005). Catalysts for hydrogen evolution from the [NiFe] hydrogenase to the Ni2P(001) surface: The importance of ensemble effect. J. Am. Chem. Soc..

[37] Grimme S (2006). Semiempirical GGA-type density functional constructed with a long-range dispersion correction. J. Computat. Chem..

[38] Delley B (2000). From molecules to solids with the DMol3 approach. J. Chem. Phys..

[39] Teimuri-Mofrad R, Rahimpour K, Ghadari R (2017). Design, synthesis and characterization of ferrocene based V-shaped chromophores with modified nonlinear effect. J. Organomet. Chem..

[40] Farhadian A (2020). A theoretical and experimental study of castor oil-based inhibitor for corrosion inhibition of mild steel in acidic medium at elevated temperatures. Corros. Sci..

[41] Verma C (2017). Corrosion inhibition of mild steel in 1M HCl by D-glucose derivatives of dihydropyrido [2,3-d:6,5-d′] dipyrimidine-2, 4, 6, 8(1H,3H, 5H,7H)-tetraone. Sci. Rep..

[42] Benzbiria N (2023). Coupling of experimental and theoretical studies to apprehend the action of benzodiazepine derivative as a corrosion inhibitor of carbon steel in 1M HCl. J. Mol. Struct..

[43] Bairagi H, Vashishth P, Sehrawat R, Shukla SK, Mangla B (2024). Impact of novel ZnO/PAA nanocomposite as corrosion inhibitor on mild steel in 5% HCl. Mater. Chem. Phys..

[44] Acidi A (2023). Examination of the main chemical components of essential oil of Syzygium aromaticum as a corrosion inhibitor on the mild steel in 0.5 M HCl medium. J. Mol. Liquids.

[45] Kandeloos AJ, Attar MM (2023). The diffusion and adhesion relationship between free films and epoxy coated mild steel. Progress Org. Coat..

[46] Mubarak G, Verma C, Mazumder MAJ, Barsoum I, Alfantazi A (2024). Corrosion inhibition of P110 carbon steel useful for casing and tubing applications in 3.5% NaCl solution using quaternary ammonium-based copolymers. J. Mol. Liquids.

[47] Shanmugapriya R (2023). Electrochemical and Morphological investigations of *Elettaria cardamomum* pod extract as a green corrosion inhibitor for Mild steel corrosion in 1 N HCl. Inorg. Chem. Commun..

[48] Banerjee S (2011). Highly efficient polyurethane ionomer corrosion inhibitor : The effect of chain structure. RSC Adv..

[49] Gnezdilov D (2024). Effective prevention of structure II gas hydrate formation using the newly synthesized kinetic inhibitors. Chem. Eng. Sci..

[50] Luo X (2024). Zwitterion modified chitosan as a high-performance corrosion inhibitor for mild steel in hydrochloric acid solution. Int. J. Biol. Macromol..

[51] Tran T-N (2024). Self-formation of protective layer on carbon steel surface in 1 M HCl solution containing *Barringtonia acutangula* leaf extract. J. Ind. Eng. Chem..

[52] Aihara J (1999). Reduced HOMO−LUMO gap as an index of kinetic stability for polycyclic aromatic hydrocarbons. J. Phys. Chem. A.

