# Supplementary material for: Prolonged corrosion protection via application of 4-ferrocenylbutyl saturated carboxylate ester derivatives with superior inhibition performance for mild steel
Source: Sci Rep. 2024 Jun 15;14:13847. doi: 10.1038/s41598-024-64471-0 (PMC11180123; doi:10.1038/s41598-024-64471-0)
Supplement: Supplementary file 1 — Supplementary Information. [file 41598_2024_64471_MOESM1_ESM.docx]

Supporting information

Prolonged corrosion protection via application of 4-ferrocenylbutyl saturated carboxylate ester derivatives with superior inhibition performance for mild steel

Hajar Jamali^a^, Saleh Moradi-Alavian^b^, Elnaz Asghari*^b^, Mehdi D. Esrafili^c^, Elmira Payami ^a^, Reza Teimuri-Mofrad*^a^

^a^ *Organic Synthesis Research Laboratory, Department of Organic Chemistry, Faculty of Chemistry, University of Tabriz, Tabriz, Iran.*

^b^ *Electrochemistry Research Laboratory, Department of Physical Chemistry, Faculty of Chemistry, University of Tabriz, Tabriz, Iran.*

*^c^ Laboratory of Theoretical Chemistry, Department of Chemistry, University of Maragheh, Maragheh, Iran.*

*Corresponding author Tel.: +98 41 33393105; Fax: +98 41 33340191.

E-mail address: [teymouri@tabrizu.ac.ir](mailto:teymouri@tabrizu.ac.ir)

*Corresponding author Tel.: +98 41 33393136; Fax: +98 41 33340191.

E-mail address: [e.asghari@tabrizu.ac.ir](mailto:e.asghari@tabrizu.ac.ir)


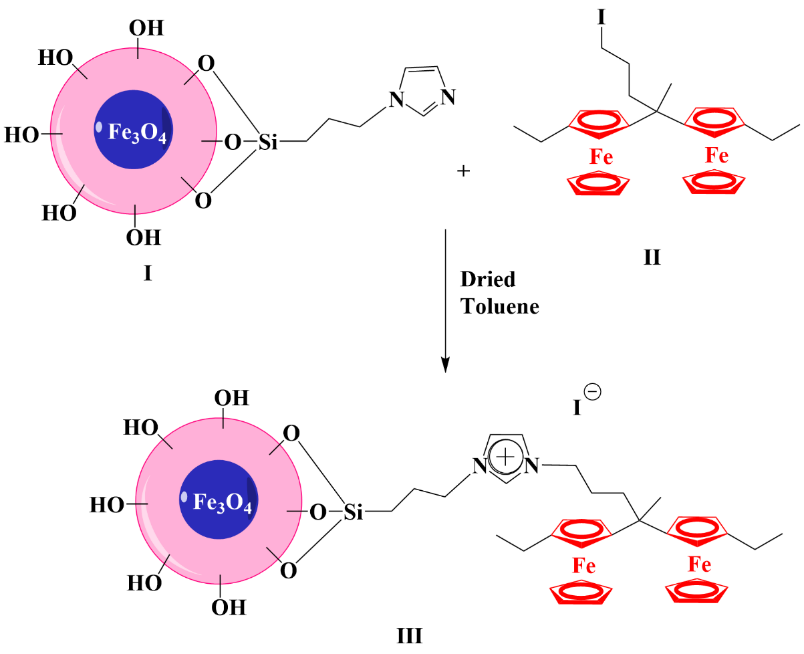


Figure S1. The synthesis of Fe_3_O_4_@SiO_2_@(CH_2_)_3_-Im-bisEthylFc[I] nanoparticles as catalyst


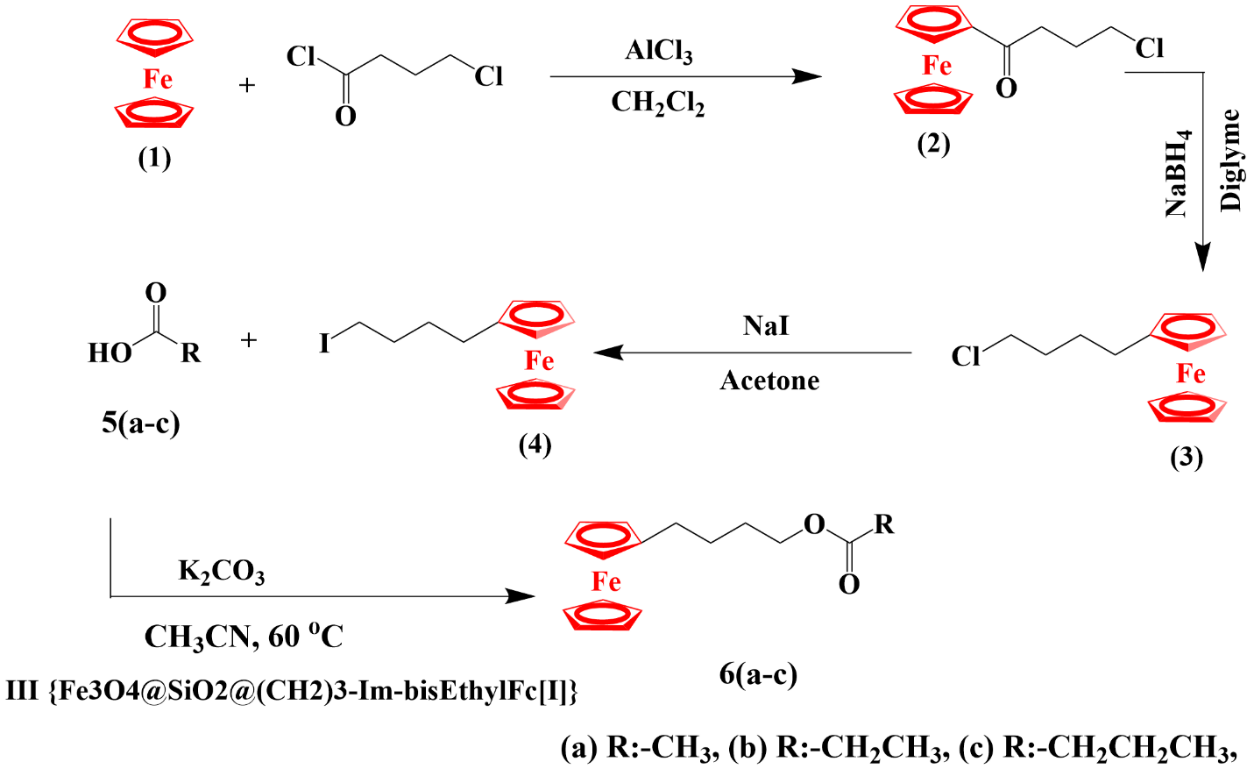


Figure S2. The synthesis route of the 4-ferrocenylbutyl saturated carboxylate esters 6a-c

|  | (a) |
| --- | --- |
|  | (b) |
| 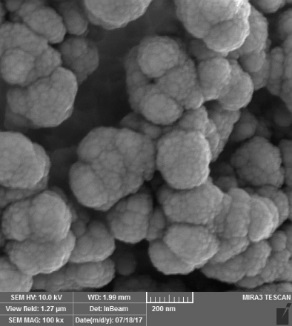 | (c) |

Figure S3. (a) The FT-IR spectra, (b) EDX and (c) SEM images of Fe_3_O_4_@SiO_2_@(CH_2_)_3_-Im-bisEthylFc[I]


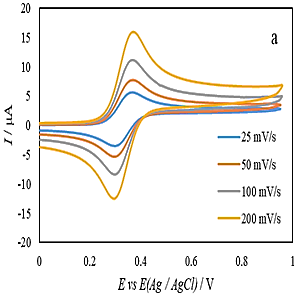

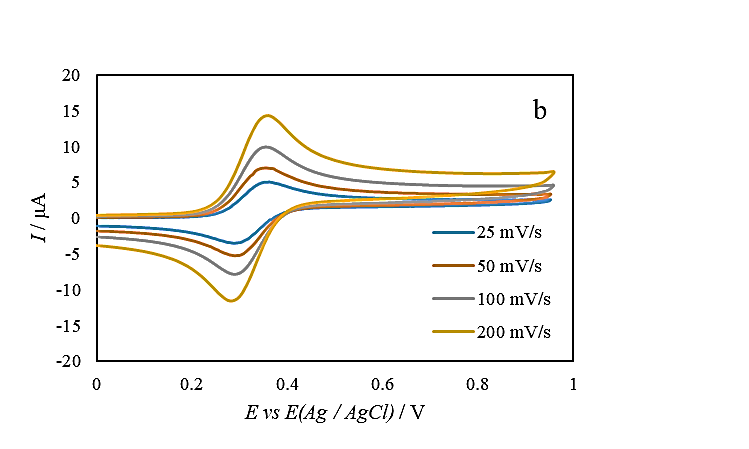

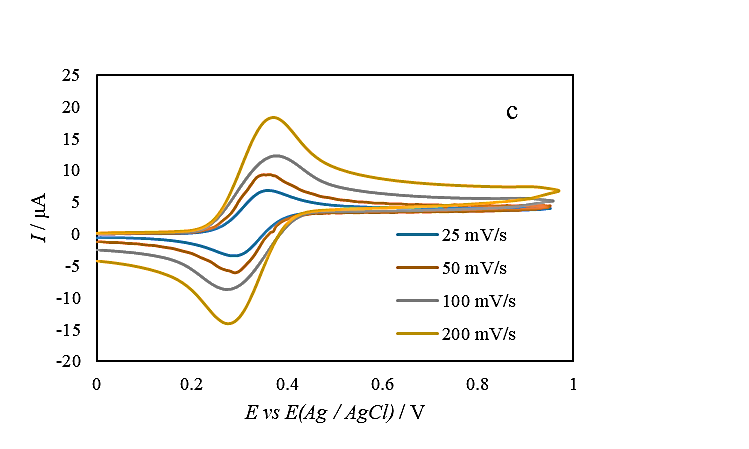


Figure S4. The CV plots of the (a) 4-ferrocenylbutyl acetate, (b) 4-ferrocenylbutyl propionate and (c) 4-ferrocenylbutyl butyrate with different scan rates.


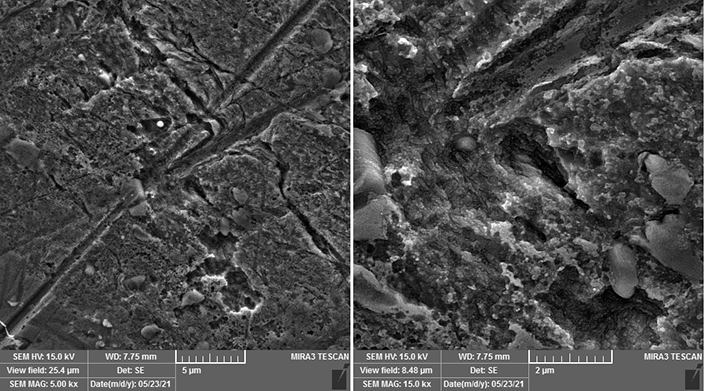


**Figure S5.** The SEM images of the mild steel surface after 24 h immersion in the corrosive acidic media with 1 M HCl at 25 °C.

Table S1. The synthesis condition and yields of 4-ferrocenylbutyl carboxylate esters with different carboxylic acids

| Product | Structure | Carboxylic acid derivatives | Solvent | Catalyst | Time (h) | T / °C | Yield (%)^a^ |
| --- | --- | --- | --- | --- | --- | --- | --- |
| **6a** |  | Acetic Acid | CH_3_CN | III | 48 | 60 | 92 |
| **6b** |  | Propionic Acid | CH_3_CN | III | 48 | 60 | 91 |
| **6c** |  | Butyric Acid | CH_3_CN | III | 48 | 60 | 93 |

a Isolated yields

Table S2. The redox parameters obtained from CV measurements of the synthesized esters from at different scan rates.

| Scan rate / mV s^-1^ | I_p,c_/μA | I_p,a_/μA | Base line corrected (I_p,a_ /I_p,c_) | (E_p,a_-E_p,c_)/mV |
| --- | --- | --- | --- | --- |
| **6a** | | | | |
| 25 | 5.5 | 3.5 | 0.63 | 73 |
| 50 | 7.6 | 5.3 | 0.70 | 71 |
| 100 | 11.1 | 8.3 | 0.75 | 70 |
| 200 | 15.8 | 12.5 | 0.79 | 65 |
| **6b** | | | | |
| 25 | 4.9 | 3.5 | 0.72 | 74 |
| 50 | 6.9 | 5.2 | 0.75 | 68 |
| 100 | 9.8 | 7.8 | 0.80 | 70 |
| 200 | 13.6 | 11.6 | 0.85 | 72 |
| **6c** | | | | |
| 25 | 6.67 | 3.39 | 0.51 | 78 |
| 50 | 9.2 | 6.1 | 0.66 | 76 |
| 100 | 12.1 | 8.6 | 0.71 | 73 |
| 200 | 17.7 | 14.0 | 0.79 | 66 |
